# Is using a consolidation tumor ratio 0.5 as criterion feasible in daily practice? Evaluation of interobserver measurement variability of consolidation tumor ratio of lung cancer less than 3 cm in size

**DOI:** 10.1111/1759-7714.14653

**Published:** 2022-10-03

**Authors:** Sachie Koike, Kimihiro Shimizu, Shogo Ide, Shuji Mishima, Shunichiro Matsuoka, Tetsu Takeda, Kentaro Miura, Takashi Eguchi, Kazutoshi Hamanaka, Taisuke Araki, Kei Sonehara, Keisuke Todoroki, Fumihito Ichinohe, Satoshi Kawakami, Masayoshi Koinuma

**Affiliations:** ^1^ Division of General Thoracic Surgery, Department of Surgery Shinshu University School of Medicine Nagano Japan; ^2^ First Department of Internal Medicine Shinshu University School of Medicine Nagano Japan; ^3^ Department of Radiology Shinshu University School of Medicine Nagano Japan; ^4^ Faculty of Pharmaceutical Sciences Teikyo Heisei University Tokyo Japan; ^5^ Center for Clinical Research Shinshu University Hospital Nagano Japan

**Keywords:** consolidation tumor ratio, interobserver variability, lung cancer classification

## Abstract

**Background:**

Consolidation tumor ratio (CTR) calculated as the ratio of the tumor consolidation diameter to the tumor maximum diameter on thin‐section computed tomography (CT) of lung cancer has been reported as an important prognostic factor. It has also been used for treatment decision‐making. This study aimed to investigate the interobserver variability of CTR measurements on preoperative CT and propose a clinically useful CTR‐based classification criterion.

**Methods:**

We enrolled 119 patients who underwent surgery for suspected or diagnosed small‐sized lung cancer (≤3.0 cm in diameter). Nine doctors reviewed preoperative CT scans to measure CTR. Interobserver variability of CTR measurements was evaluated using the coefficient of variation (CV) and Fleiss' *κ*. The prognostic effect of the CTR‐based classification was assessed using the Kaplan–Meier method.

**Results:**

Interobserver variability of CTR measurement was the highest for tumors with the lowest CTR (CTR = 0); it decreased as CTR increased and reached a plateaued level of low variability (CV <0.5) at CTR of 0.5. We proposed a three‐group classification based on the findings of CTR interobserver variability (CTR < 0.5, 0.5 ≤ CTR < 1, and CTR = 1). Interobserver agreement of the judgment of the CTR‐based classification was excellent (Fleiss' *κ* = 0.81). The classification significantly stratified patient prognosis (*p* < 0.001, 5‐year overall survival rates with CTR < 0.5, 0.5 ≤ CTR < 1, and CTR = 1 were 100, 88, and 73.8%, respectively).

**Conclusions:**

CTR 0.5 is a clinically relevant and helpful cutoff for treatment decision‐making in patients with early‐stage lung cancer based on high interobserver agreement and good prognostic stratification.

## Graphical abstract

Interobserber variability of consolidation tumor ratio (CTR) lesser in CTR ≥ 0.5 lung cancer (a), and interobserber agreement of CTR < 0.5, CTR ≥ 0.5, CTR 1.0 diagnosis was excellent (b). Over all survival rate reduced with CTR increase (c). CTR 0.5 is favorable cutoff with good interobserver agreement and prognosis prediction.

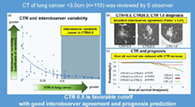

## INTRODUCTION

The detection of lung cancer has increased with the introduction of computed tomography (CT), especially in the small‐sized lung cancer with ground glass opacity (GGO).^1^ The findings from thin‐section CT of small‐sized lung cancer have been reported to be the best predictors of pathologic invasiveness, and the prognostic effect of GGOs or solid components on thin sections has been studied.^2^

Several studies have revealed that consolidation tumor ratio (CTR) and solid component size on thin‐section CT are more important prognostic factors than maximum tumor size.^2^, ^3^, ^4^, ^5^, ^6^, ^7^ The Japan Clinical Oncology Group (JCOG) 0201 study investigated the appropriateness of CTR (0.5) for cT1a‐b (≤3.0 cm) and 0.25 for cT1a (≤2.0 cm) by comparing CTR and pathological findings of resected specimens. They reported that radiological noninvasive adenocarcinoma could be defined as an adenocarcinoma ≤2.0 cm with CTR ≤ 0.25.^2^ Subsequently, the eighth edition of the tumor‐node‐metastasis (TNM) staging system states that clinical T should be based on the size of its solid component.^8^ The results from these reports suggest that measuring CTR and the size of the tumor solid component on preoperative CT may have great clinical value in the prognosis of patients with lung cancer. However, in daily practice, it is sometimes difficult to measure the size of consolidation or CTR. Therefore, interobserver variability may increase, particularly in part‐solid nodules with GGO.

This study aimed to investigate the interobserver variability in the measurement of CTR of small‐sized lung cancer and evaluate the feasible CTR classification criteria (for example, “larger than CTR 0.5 or not” or “larger than CTR 0.25 or not”) in daily practice. Additionally, we studied the relationship between CTR and the prognosis of our patients and evaluated the classification criteria based on prognosis.

## METHODS

### Patients

This study was approved by the ethics review board of Shinshu University School of Medicine, Matsumoto, Japan (approval no. 4738), and the requirement for informed patient consent was waived.

In total, 539 patients with suspected or diagnosed lung cancer underwent surgery at Shinshu University Hospital between January 2010 and December 2014. Among these, 119 out of 387 patients with tumors ≤3.0 cm in diameter on preoperative CT were randomly selected and enrolled in this study. Patients who had undergone prior lung resection or had lymph node metastasis were excluded (Figure S1).

### Radiological evaluation

We evaluated preoperative CT scans of enrolled patients with a section thickness of 0.63–1.25 mm. If a patient had multiple lesions, one lesion was designated for evaluation. We chose three thoracic surgeons (with 5‐, 10‐, and 14 years of experience), two respiratory physicians (with 9‐ and 11‐years of experience), two radiologists (with 8‐ and 25‐years of experience), and two surgical residents (with 2‐ and 3 years of experience) as observers. Each observer independently measured the following parameters: (1) maximum diameter of the tumor, (2) maximum diameter of the consolidation part of the tumor, and (3) tumor CTR calculated from (1) and (2). The measurement was performed using a lung window setting (window level −550 Hounsfield units; window width 1500 Hounsfield units). To mirror daily practice, observers could evaluate the tumors in any orientation (axial, sagittal, or coronal). If the observers could not evaluate the consolidation diameter (for example, when the tumor consolidation part exceedingly mixed with the GGO part), they classified the tumor as “unable to diagnose” and excluded the evaluation from the analysis.

The “gold standard” of CTR was determined by two surgeons (Sachie Koike, first author, and Kimihiro Shimizu, corresponding author) blinded to patient clinical and pathological outcomes. Two surgeons measured CTR together and determined the final status of CTR as the gold standard. We calculated CTR to four decimal places and rounded to three decimal places when categorizing the gold standard CTR 0.05. We also evaluated the relationship among mean diameter, mean value of the solid component diameter, and interobserver variability.

### Classification of the types of tumors

We classified 119 enrolled tumors into six types based on the criteria proposed by Suzuki et al.^9^ The criteria are shown below and in Figure S2.

Type 1: Pure (simple) GGO

Type 2: Semi consolidation (an area of intermediate homogeneous increase in density)

Type 3: Halo (area comprising solid part and surrounding GGO halo)

Type 4: Mixed (an area comprising GGO and solid part with air bronchogram)

Type 5: Solid pattern with GGO (area of GGO should be less than 50%)

Type 6: Solid pattern

Type 1 to 4 included tumors where less than 50% was solid, whereas types 5 and 6 included those where more than 50% was solid. We compared interobserver variability among different types of tumors. Tumor classification was performed by two thoracic surgeons (Sachie Koike and Kimihiro Shimizu).

### Classification criteria “CTR < 0.5, 0.5 ≤ CTR < 1, or CTR = 1 (pure solid)”

We hypothesized that interobserver variability of classification, whether CTR ≥ 0.5 or not, might be relatively small, and we created the tumor classification criteria “CTR < 0.5, 0.5 ≤ CTR < 1, or CTR = 1 (pure solid)” and studied interobserver agreement of diagnosis based on whether the tumors were CTR < 0.5, 0.5 ≤ CTR <1, or CTR = 1 (pure solid) to evaluate the feasibility of the criteria. Some observers could not measure CTR of some tumors such as the mixed type because the consolidation part of the tumors was mixed with GGO. We categorized these diagnosis as “unable to diagnose” and excluded them from the analysis. In addition, we classified the tumors into three groups based on the gold standard of CTR determined by two surgeons (Sachie Koike and Kimihiro Shimizu) as described above and evaluated the overall survival (OS) of each group to estimate the probability of survival.

### Statistical analysis

Interobserver variability in measuring the diameter of tumors, diameter of the consolidation part of tumors, and CTR were determined using the coefficient of variation (CV). First, the differences among the observers with respect to tumor diameter, consolidation diameter, and CTR measurements were calculated as standard deviation (SD). CV was calculated as the SD to the mean (SD/mean) ratio. Although SD represents the dispersion of measurement variability, it is affected by the mean value. CV was calculated to correct the difference in the mean value.

Interobserver agreement of the diagnosis whether the tumors were CTR < 0.5, 0.5 ≤ CTR < 1, or CTR = 1 (pure solid) was evaluated with Fleiss' *κ* statistics. Fleiss' *κ* statistics measure the interobserver agreement of more than three observers.^9^ Fleiss' *κ* was categorized as poor (0 < κ/κw ≤ 0.20), fair (0.20 < κ/κw ≤ 0.40), moderate (0.40 < κ/κw ≤ 0.60), good (0.60 < κ/κw ≤ 0.80), and excellent (0.80 < κ/κw ≤ 1.00).^10^, ^11^

The probability of survival of patients in the CTR < 0.5, 0.5 ≤ CTR <1, and CTR = 1 (pure solid) groups was estimated using the Kaplan–Meier method, and survival curves were drawn. OS was defined as the time from tumor resection to death (from any cause) with patients still alive being censored at last follow‐up. Survivors were censored from the analysis at the time of the last follow‐up, regardless of disease status. The differences in survival among the groups were tested using the log‐rank test.

Characteristic differences between the three groups were assessed using Kruskal–Wallis test for continuous variables and the Chi‐squared and Fisher's exact tests for categorical variables.

Differences were considered statistically significant at *p* < 0.05. All statistical analyses were performed using SPSS statistical software (version 26.0; SPSS, IBM).

## RESULTS

### Clinicopathological characteristics

The clinicopathological characteristics of the patients are shown in Table 1. Among the included patients, 55 had CTR <0.5, 35 had 0.5 ≤ CTR <1, and 29 had CTR = 1 (pure solid). There were significant differences among the three groups with respect to clinicopathologic factors (*p* < 0.05 was observed for almost all factors). As the CTR increased, the number of smoking patients increased, the pathological T stage increased, and the proportion of adenocarcinoma decreased.

**TABLE 1:** Patient characteristics

Factors	CTR < 0.5 (*n* = 55)	0.5 ≤ CTR < 1.0 (*n* = 35)	CTR = 1.0 (*n* = 29)	*p*‐value
Sex (male)	20 (36.4)	13 (37.1)	27 (93.1)	<0.001
Age (years)	66 (61–73)	65 (60–76)	74 (68–77.5)	0.073
Smoking	21 (38.2)	16 (45.7)	23 (79.3)	0.001
Operative procedure				0.004
Wedge	14 (25.5)	7 (20.0)	3 (10.3)	
Segmentectomy	14 (25.5)	1 (2.9)	2 (6.9)	
Lobectomy	27 (49.1)	27 (77.1)	24 (82.8)	
Pathological T stage				<0.001
pTis/T1mi	26 (47.3)	7 (20.0)	0 (0.0)	
pT1a	17 (30.9)	5 (14.3)	1 (3.4)	
pT1b	9 (16.4)	14 (40.0)	13 (44.8)	
pT1c	0 (0.0)	4 (11.4)	8 (27.5)	
>pT2	3 (5.4)	5 (14.3)	7 (24.1)	
Histology				<0.001
Adenocarcinoma	54 (98.2)	34 (97.1)	11 (37.9)	
AIS/MIA	23 (41.8)	4 (13.8)	0 (0.0)	
Lepidic	7 (12.7)	5 (14.3)	1 (3.4)	
Others	1 (1.8)	1 (2.9)	18 (62.0)	
Adenosquamous	1		1	
Pleomorphic		1	1	
LCNEC			3	
SCC			13	

*Note*: Categorical data are shown as *n* (%) and continuous data as median, range, 25%–75% interquartile range.

Abbreviations: Adenosquamous, adenosquamous carcinoma; AIS, adenocarcinoma in situ; CTR, consolidation tumor ratio; LCNEC, large cell neuroendocrine carcinoma; Lepidic, lepidic predominant adenocarcinoma; MIA, minimally invasive adenocarcinoma; Pleomorphic, pleomorphic carcinoma; SCC, squamous cell carcinoma.

### Interobserver variability of CTR, tumor size, and solid component size

**FIGURE 1:**
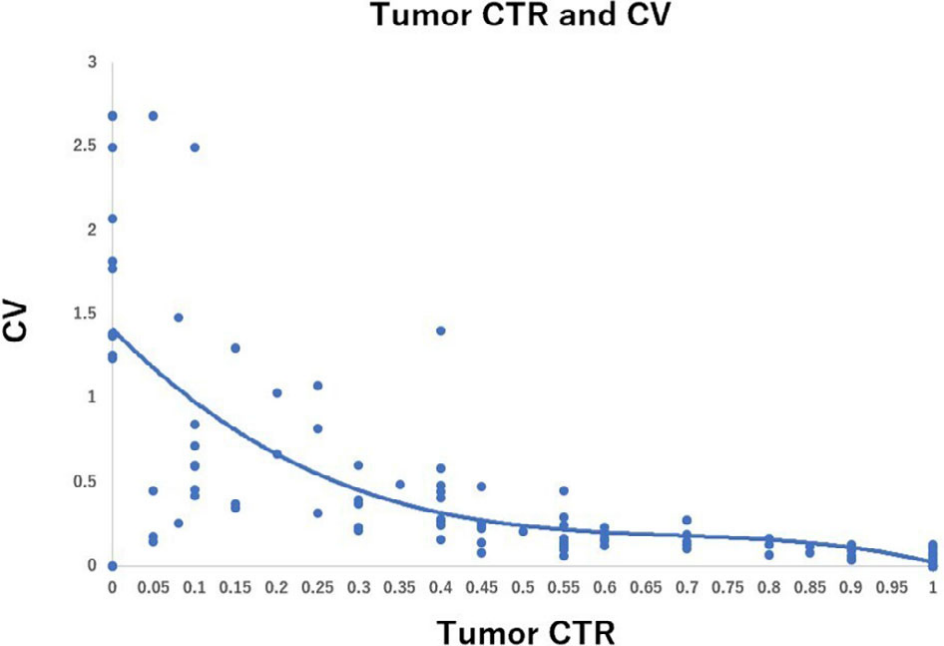
Relationship between tumor consolidation tumor ratio (CTR) and coefficient of variation (CV)

**FIGURE 2:**
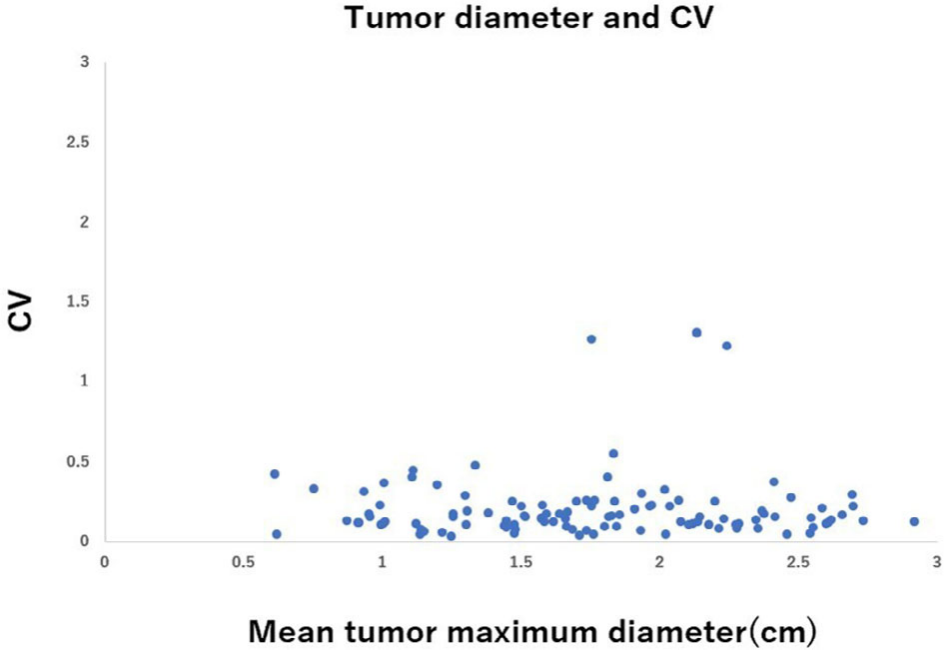
Relationship between tumor mean maximum diameter and coefficient of variation (CV)

**FIGURE 3:**
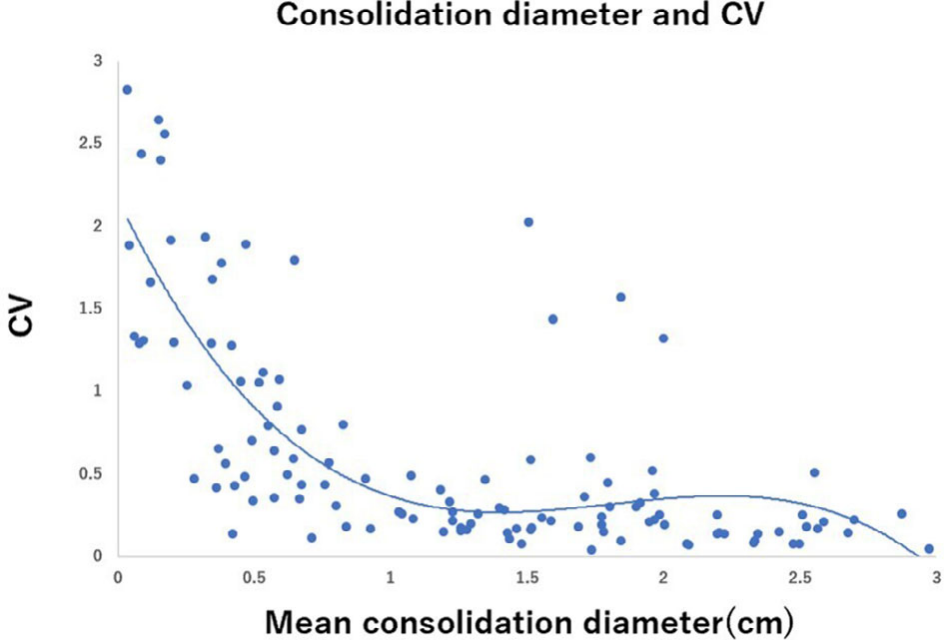
Relationship between tumor mean consolidation diameter and coefficient of variation (CV)

The relationship between the CTR of tumors and the CV of CTR measurements is shown in Figure 1. The figure suggests that the interobserver variability (CV) in CTR measurement was greater in tumors with lower CTR, such as pure GGO or CTR ≤ 0.25 tumors. It decreased as the CTR increased and reached a plateaued level of low variability at CTR = 0.5. Figure 2 shows the relationship between mean tumor maximum diameter and CV. CV was lower than 0.5 in almost all tumors, and interobserver variability in the measurement of tumor size was relatively small, regardless of the tumor size. The relationship between the mean solid component size of the tumor and the CV is shown in Figure 3. Interobserver variability in the measurement of the solid component size of the tumors was greater in tumors with smaller solid component sizes. It decreased as the solid component size increased and reached a plateaued level of low variability at 1.5 cm.

### Interobserver variability comparison among tumor types

**FIGURE 4:**
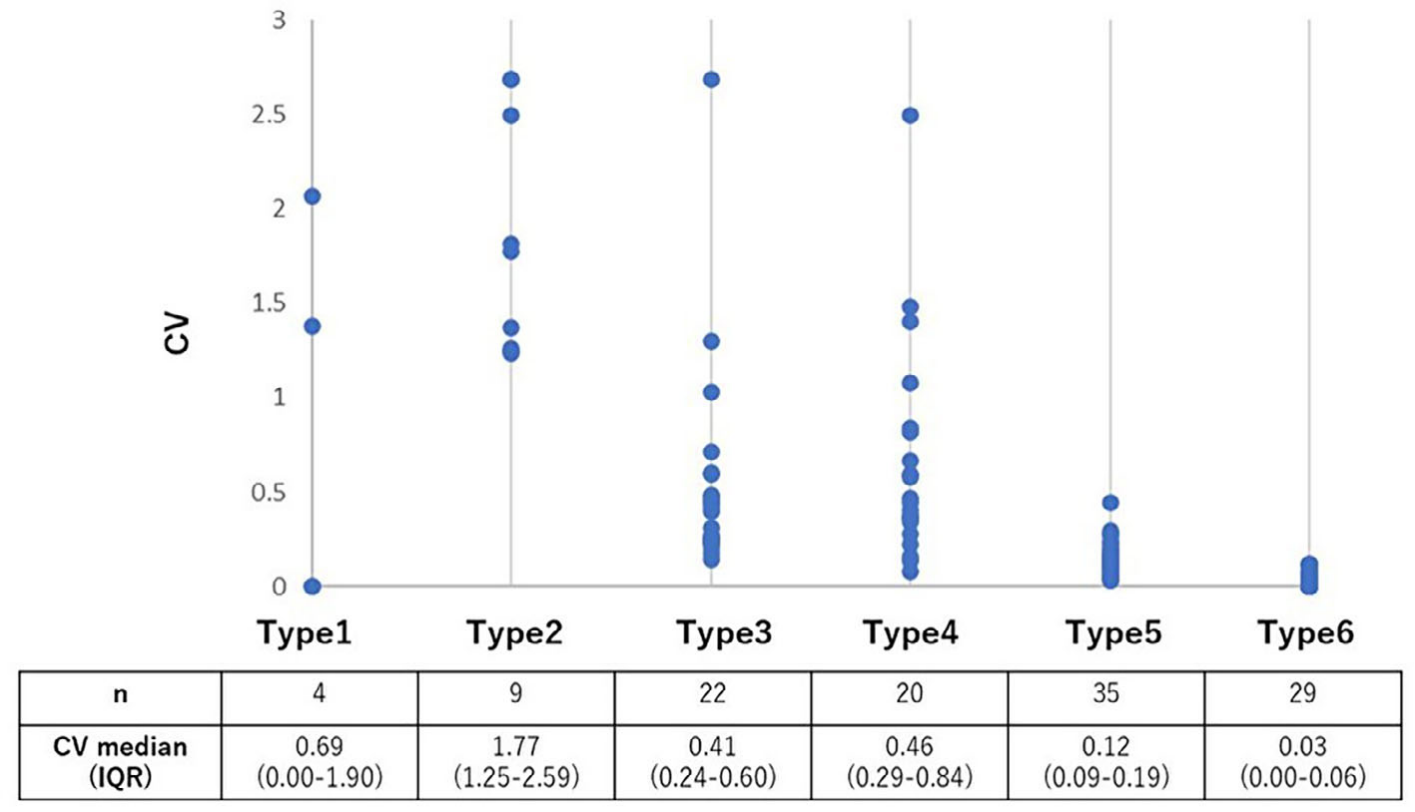
Relationship between tumor type and coefficient of variation (CV) of consolidation tumor ratio (CTR) measurement. *n*, number of patients; IQR, interquartile range

We classified 119 tumors into six types according to the criteria proposed by Suzuki et al.^1^ Four tumors were classified as type 1 (pure GGO), nine as type 2 (semiconsolidation), 22 as type 3 (halo), 20 as type 4 (mixed), 35 as type 5 (solid pattern with GGO), and 29 as type 6 (solid pattern). The median CV (interquartile range) of CTR measurements was the highest for type 2 (median [25–75 percentiles], 1.77 [1.25–2.59]) followed by type 1 (0.69 [0.00–1.90]), type 4 (0.46 [0.29–0.84]), type 3 (0.41 [0.24–0.60]), type 5 (0.12 [0.09–0.19]), and type 6 (0.03 [0.00–0.06]) (Figure 4).

### Interobserver agreement of the classification criteria “CTR < 0.5, 0.5 ≤ CTR < 1, or CTR = 1 (pure solid)”

The ratio of diagnosis different from golden standard (different diagnosis) in the CTR < 0.5 group was 20.5% (total diagnoses, 462; different diagnoses, 95; unable to diagnose, 33), in the 0.5 ≤ CTR < 1 group was 3.0% (total diagnoses, 297; different diagnoses, 9; unable to diagnose, 18), and in the CTR = 1 group was 6.9% (total diagnoses, 247; different diagnoses, 17; unable to diagnose, 14). The Fleiss' *κ* of the interobserver agreement of CTR <0.5, 0.5 ≤ CTR <1, and CTR = 1 (pure solid) diagnosis was 0.81, and the extent of the agreement was excellent (Table 2).

**TABLE 2:** Observers judgment and agreement

Tumor CTR	*n*	Different /all (%)
CTR <0.5	55	95/462 (20.5)
0.5 ≤ CTR < 1	35	9/297 (3.0)
CTR = 1	29	17/247 (6.9)
Fleiss' *κ* = 0.81		

Abbreviations: CTR, consolidation tumor ratio; Different, different diagnosis from golden standard.

### Prognostic analysis

**FIGURE 5:**
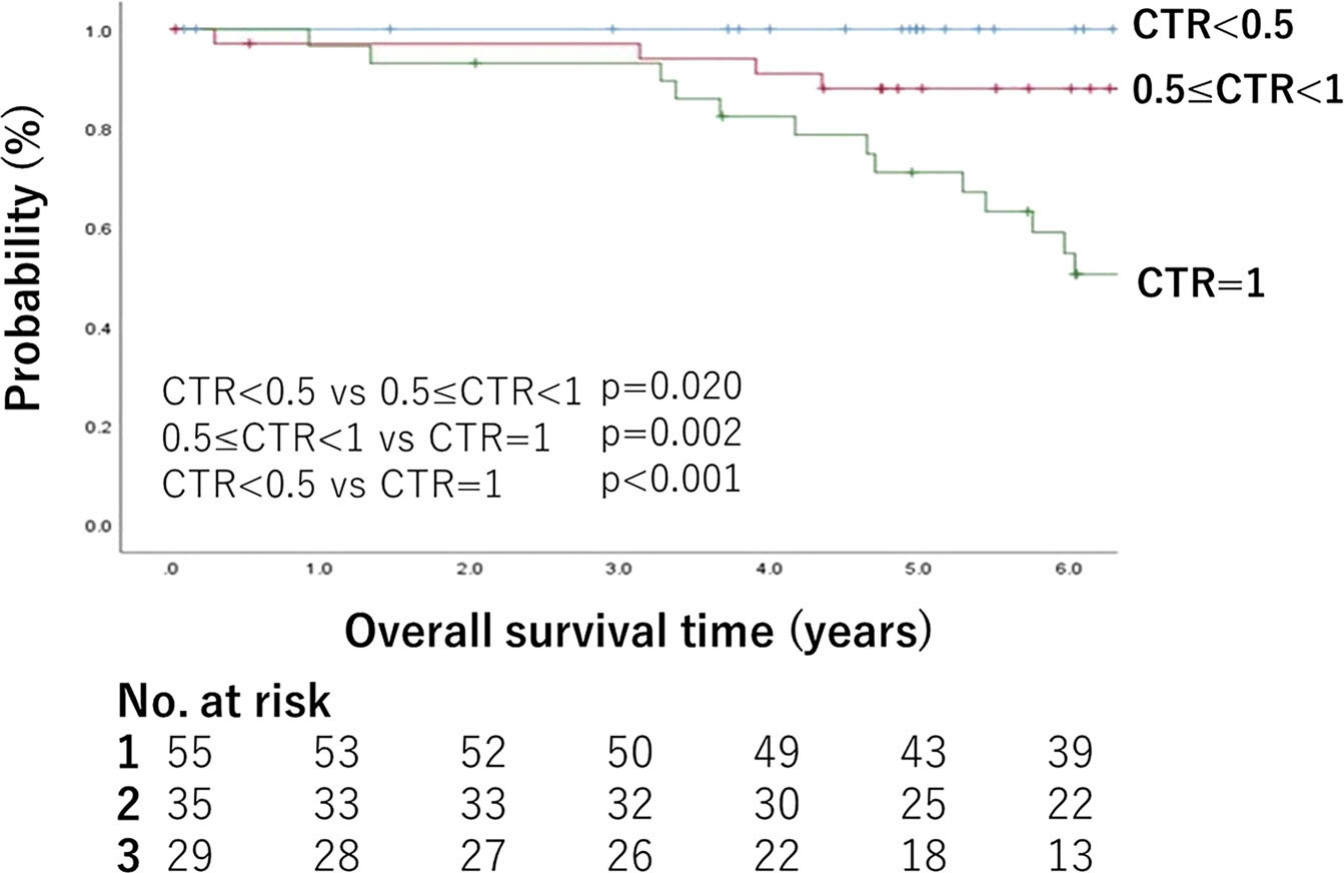
Overall survival (OS) curves of 119 patients are presented based on tumor consolidation tumor ratio (CTR): <0.5 (blue line); ≥0.5 (red line); and 1.0 (green line)

Death after tumor resection was observed in 20 patients (16.8%). Figure 5 shows the Kaplan–Meier survival curves. The 5‐year OS rate was 100% (95% confidence interval [CI], 100.0–100.0) in the CTR < 0.5 group, 88.0% (95% CI: 76.9–99.0) in the 0.5 ≤ CTR < 1 group, and 73.8% (95% CI: 56.9–90.5) in the CTR = 1 group (number of lost to follow‐up; CTR < 0.5:15, CTR ≥ 0.5:9, CTR 1.0: 4). The median follow‐up period was 79.2 months. The difference in survival rates among these groups was statistically significant (all: *p* < 0.001, CTR <0.5 vs. 0.5 ≤ CTR <1: *p* = 0.02, 0.5 ≤ CTR < 1 vs. CTR = 1, *p* = 0.002).

## DISCUSSION

This study investigated interobserver variability in CTR measurements and proposed a CTR‐based classification. The novelty and strengths of this study are as follows: (1) we found a clinically relevant cutoff of CTR (CTR = 0.5) based on interobserver variability; (2) we proposed a new three‐group classification using the cutoff of CTR 0.5 (CTR < 5, 0.5 ≤ CTR < 1, CTR = 1); and (3) our proposed three‐group classification showed excellent interobserver reliability and good prognostic stratification.

In our study, the interobserver variability of CTR measurement decreased as CTR increased and reached a plateaued level of low variability at CTR = 0.5. The results suggest that a cutoff of CTR <0.5 (such as CTR 0.25, etc) is not an appropriate cutoff with great interobserver variability, and CTR = 0.5 is the best cutoff value. Similar to our study, Nair et al. studied 107 radiologists' CT results of 69 lung nodules (solid, part‐solid, or ground glass) and investigated the relationship between the median and CV of the solid proportion. The study reported a strong negative correlation (Spearman's rank correlation coefficient = −0.88, *p* < 0.0001).^11^ The results suggested that when the solid proportion of lung nodules (CTR) decreased, the interobserver variability in the measurement of the solid proportion increased. This observation supports our results. We also studied interobserver variability in the measurement of tumor size and consolidation size of tumors. The interobserver variability was relatively low in all size of tumors (CV ≤ 0.5 in most of the tumors). However, the interobserver variability while measuring the consolidation size was greater in smaller consolidation size tumors (pure GGN or CTR ≤ 0.25 tumors) (Figures 2 and 3), suggesting that interobserver variability may lead to variations in CTR measurements.

We also evaluated the effect of morphological characteristics of tumors on interobserver variability using radiological classification criteria of small adenocarcinoma of the lung reported by Suzuki et al. They classified lung adenocarcinoma 2 cm or less into six groups; types 1, 2, 3, and 4 were radiologically confirmed to be early adenocarcinomas of the lung, and their pathological features were minimally invasive.^9^ We compared interobserver variability in the measurement of CTR for each of the six tumor types and found greater interobserver variability in pure GGO (type 1) and semiconsolidation (type 2) tumors. Additionally, a relatively greater interobserver variability was demonstrated in the halo (type 3) and mixed type (type 4) tumors. These results suggest that regardless of the morphological characteristics of tumors, tumors with CTR < 0.5 had great interobserver variability during CTR measurement.

Our group previously investigated the interobserver variability of six observers in evaluating the lung tumor diameter of 47 patients on preoperative CT. The study reported that two experienced observers measured relatively smaller diameters than other observers, probably because of their increased ability to efficiently discriminate blood vessels, bronchi, atelectasis, and inflammatory changes from the tumor compared to other observers.^12^ As stated above, the ability to diagnose blood vessel/bronchi/atelectasis/inflammation or consolidation part of the tumor varies among observers. These variations may have a greater effect when evaluating pure GGO/semiconsolidation and CTR ≤ 0.25 tumors, possibly leading to the observation that interobserver variability of measuring CTR or tumor solid component is greater in smaller CTR tumors such as pure GGO, semiconsolidation lesions, and CTR < 0.5 part‐solid nodules.

To summarize the result of interobserver variability in our study, tumors with CTR < 0.5 demonstrate great interobserver variability of CTR measurement despite tumor types (or morphological characteristics), and dividing these tumors into further subgroups (CTR < 0.25 and 0.25 ≤ CTR < 0.5) might not be reasonable. Furthermore, tumors with solid component smaller than 1.5 cm may have great interobserver variability. This may indicate that the diagnosis of cT1a and cT1b in GGO tumors may have great interobserver variability. In contrast, tumors with CTR ≥ 0.5 demonstrated relatively small interobserver variability. Based on these facts, we created the tumor classification criteria based on CTR as “CTR < 0.5, 0.5 ≤ CTR < 1, or CTR = 1 (pure solid).” The interobserver agreement of the criteria was excellent (Fleiss' *κ* = 0.81), proving the feasibility of the criteria from the aspects of interobserver agreement. We investigated the survival of patients in the CTR < 0.5, 0.5 ≤ CTR < 1, or CTR = 1 (pure solid) tumor groups, and it was significantly different among the groups (all: *p* < 0.001, CTR < 0.5 vs. 0.5 ≤ CTR <1, *p* = 0.02, 0.5 ≤ CTR < 1 vs. CTR = 1: *p* = 0.002). These results suggest that the classification criteria “CTR < 0.5, 0.5 ≤ CTR < 1 or 1 (pure solid)” could be favorable prognostic predictors with small interobserver variability, as the clinical T category in TNM classification. Hattori et al. divided stage IA lung cancers into GGO and solid (without GGO) groups and reported that the 5‐year OS was significantly different between the two groups (95.1% vs. 81.1%). They concluded that the presence or absence of GGO (CTR < 1.0 or CTR 1.0) should be considered an important parameter in the next clinical T classification.^13^ In addition, Obayashi et al. reported that the tumor volume doubling time (VDT) of primary lung cancer was significantly longer in adenocarcinoma with GGO components than those without such components (median VDT: 725 and 177 days, respectively).^14^ These reports support the feasibility of classifying small lung cancer into 0 ≤ CTR < 1.0 and CTR 1.0 (pure solid) groups. Our study divided the 0 ≤ CTR <1.0 group (with GGO group) into CTR < 0.5 and 0.5 ≤ CTR < 1, and the 5‐year OS was significantly different between these subgroups. Similar to our study, the JCOG0201 study defined CTR of 0.5 or less in <3.0 cm tumors as noninvasive tumors with an excellent prognosis.^2^ In contrast to the JCOG0201 study and our study, Hattori et al. reported that the CTR difference in part‐solid nodules did not affect the 5‐year OS.^15^ They estimated the reason for this result to be the high frequency of the lepidic component in part‐solid tumors, which could be completely controlled by surgical resection. In our study, the frequency of the lepidic component in CTR ≥ 0.5% tumors was lower than that in Hattori's report (14.3% vs. 23.0%). The difference in pathological lepidic components might have caused the differences in the prognostic results of the studies.

Currently, sublobar resections, such as segmentectomy and wedge resection, have been reported to be effective for GGO‐dominant peripheral small‐sized lung cancer instead of conventional standard surgical procedures such as lobectomy.^16^, ^17^ The JCOG 1211 study^18^ is currently evaluating the outcomes of segmentectomy for adenocarcinoma ≤3.0 cm with CTR ≤ 0.5. The results of our study revealed the feasibility of lung cancer classification using CTR 0.5 in tumors ≤3.0 cm from the aspect of interobserver agreement and prognosis. This result may support the concept of the JCOG1211 study and predict a good outcome.

This study had several limitations. First, the number of patients was relatively small. Second, this was a single‐institution study. Third, the number of observers was only nine, which was relatively small. Fourth, the career and specialty of observers varied, but they could mirror daily practice. Fifth, the golden standard of tumor CTR and tumor classification were determined by two thoracic surgeons. Finally, the patients enrolled in this study were negative for lymph node metastasis.

In conclusion, lung cancer classification criteria “CTR < 0.5, 0.5 ≤ CTR < 1, or CTR = 1 (pure solid)” might be favorable criteria in terms of interobserver agreement and prognosis prediction. These criteria would be an important factor in considering the next clinical T classification.

## CONFLICT OF INTEREST

There are no conflicts of interest to declared.

## Supporting information


Appendix S1

**Figure S1** Flow chart of patients. Between January 2010 and December 2014, 539 patients with suspected or diagnosed lung cancer underwent surgery at Shinshu University Hospital. A total of 387 patients with tumors ≤3.0 cm in diameter were enrolled in this study. Patients who had undergone prior lung resection or had lymph node metastasis were excluded. A total of 119 of 387 patients were randomly selected for radiological evaluation

**Figure S2** Lung cancer type classification criteria proposed by Suzuki et al. Type 1: pure GGO, Type 2: Semiconsolidation, Type 3: Halo, Type 4: Mixed, Type 5: Solid with GGO, Type 6: Solid. The consolidation component of Types 1–4 is less than 50%, and Type 5, 6 is more than 50%. GGO, ground‐glass opacity
