# Supplementary material for: Is using a consolidation tumor ratio 0.5 as criterion feasible in daily practice? Evaluation of interobserver measurement variability of consolidation tumor ratio of lung cancer less than 3 cm in size
Source: Thorac Cancer. 2022 Oct 3;13(21):3018–24. doi: 10.1111/1759-7714.14653 (PMC9626346; doi:10.1111/1759-7714.14653)
Supplement: Supplementary file 1 — Appendix S1 [file TCA-13-3018-s001.docx]

**Figure S1.** Flow chart of patients. Between January 2010 and December 2014, 539 patients with suspected or diagnosed lung cancer underwent surgery at Shinshu University Hospital. A total of 387 patients with tumors ≤3.0 cm in diameter were enrolled in this study. Patients who had undergone prior lung resection or had lymph node metastasis were excluded. A total of 119 of 387 patients were randomly selected for radiological evaluation.

**Figure S2.** Lung cancer type classification criteria proposed by Suzuki et al. Type 1: pure GGO, Type 2: Semiconsolidation, Type 3: Halo, Type 4: Mixed, Type 5: Solid with GGO, Type 6: Solid. The consolidation component of Types 1-4 is less than 50%, and Type 5, 6 is more than 50%.

GGO=ground glass opacity
